# Piccadilly full of people and other foul things: On heroism and perversion in the post-pandemic era

**DOI:** 10.1038/s44319-024-00143-4

**Published:** 2024-04-26

**Authors:** Vladimir Leksa

**Affiliations:** Laboratory of Molecular Immunology, Institute of Molecular Biology, Slovak Academy of Sciences, Dubravska cesta 21, 845 51 Bratislava, Slovakia

**Keywords:** Economics, Law & Politics, History & Philosophy of Science

## Abstract

Science gifts us the tools to solve the pressing humanitarian and ecological challenges of our time. But ignorance and selfishness prevent us from using science to the best effect.

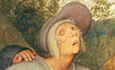

Sometime during the Covid-19 pandemic I noticed certain changes in my behaviour. For example, when someone was approaching me on the sidewalk, I switched to the other side of the street. Or when I saw a cluster of, say, two or more citizens, I avoided them all together. Step by step, I happened to be like a certain aristocrat from the short story *Mulliner Nights* by Sir Pelham Grenville Wodehouse, who described his walk in London by a laconic notion that “Piccadilly was full of people and other foul things.” I asked myself if my behaviour was heroic or rather coward.

Inasmuch as there are opposite approaches. One old man in Edgar Allan Poe’s short story *The man of the crowd* was looking for the busiest places in the city unceasingly, where there was the greatest noise, the thickest hustle and bustle. Early in the morning, he blended into the groups of milkmen rushing to work; during the day, he wandered aimlessly among bank clerks and passengers at the stations; in the evening, he occurred in bars; and after the closing time, he rambled the streets in the heart of the city till the next morning. Poe characterized the figure as follows: “This old man is the type and the genius of deep crime. He refuses to be alone”.

During the pandemic, many people found themselves within manners not very different. After all, isn’t constant engagement to social networking just a refusal to be alone? I asked myself whether this behaviour is really criminal as Poe had it or rather heroic. Who was the hero then, during the pandemic, and who was the genius of crime? And which is who actually now, in the post-pandemic era?

## The heroic age of science

When I was a schoolboy, I had a list of heroes. I remember that Bud Spencer, Niki Lauda, Terry Fox, Cyrus Smith and John Lennon were among them. As I was growing up and getting educated, more and more scientists joined the list. Take, for example, Louis Pasteur. He was the first to formulate the theory that infectious diseases are caused by pathogenic microorganisms (Brey, 2022). His theory was outright dismissed and Pasteur even became a laughing stock, especially among his fellow academics who were convinced they knew better. However, Pasteur was not discouraged. He observed disease-causing bacteria by an optical microscope, but he was not able to identify the causative agent of rabies. Yet, he did not abandon his theory. He showed the same humility as Mendeleev who did not throw away his table of elements when the elements known at that time did not exactly fit into it. Instead, he accepted the possibility that not all elements were known and left empty spots in his chart for the unknown ones. In short, he was not convinced that he already knew everything.

Likewise, Pasteur concluded that the optical technology of the time did not yet allow him to see the pathogen causing the deadly rabies. He was right, this only became possible in 20th century after the invention of the electron microscope: it was a virus, thousand times smaller than a bacterium. Pasteur fought against the invisible killer successfully—he was the first to prepare a vaccine against rabies that saved millions of lives up-to-now. Today, the Pasteur Institute in Paris is one of the most prestigious scientific institutions in the world, and Pasteur is regarded as a hero.

There have been countless stories like Pasteur’s in the history of science. At the dawn of immunology, Edward Jenner invented a vaccine against smallpox, which until then had been one of the greatest killers of humankind (Esparza, 2020). Jenner proved that a vaccine obtained from the blisters of milkmaids who had just overcome cowpox, harmless to humans, would protect us from the deadly species. He did not escape ridicule either. Cartoons of Jenner crowned with cow horns mocked his work. It did not last long till the cartoons were replaced by statues created out of gratitude and respect. Today, smallpox is eradicated, and Jenner is also seen as a hero.

Ignaz Philipp Semmelweis came off worse (Kadar, 2021). The Hungarian doctor working in the Vienna General Hospital was troubled by how many young mothers died of puerperal fever after giving birth. Like Pasteur, he assumed that their deaths were caused by microorganisms most likely transmitted by his fellow doctors, who often came to the delivery rooms straight from the dissecting rooms. Semmelweis appealed to them to wash their hands before approaching women in labour. Nothing more. Nevertheless, far-famed imperial-royal physicians felt so much offended by his advice that instead of their hands they treated their colleague like dirt. Semmelweis ended up with severe depression in an asylum, where he later died—of an infection. It was only after his death, when doctors started to wash their hands and the dangerous puerperal fever disappeared from the maternity wards.

Another heroic act from Vienna was not tragic at all. In the first half of the 19th century, Viennese drank the Danube water, very much the same they defecated into. And they often died of typhoid fever or cholera. Until, based on the project of the geologist Eduard Suess (Sengör and Dullo, 2021), they built an aqueduct to deliver pure mountain water from under the Alpine Rax directly to Vienna. The deadly epidemics disappeared from the emperor’s city the very next days.

Throughout history, achievements of science and technology have saved human lives and we justly consider their authors as heroes and heroines nowadays. Three or four years ago, we went through something that humankind had not experienced since the dawn of modern history—right in the middle of a global pandemic, we battled the deadly virus with a new vaccine. However, the vaccine did not come out of the blue, it was the result of long-term patient scientific work complicated by oblivion and dismissal similar to those once experienced by Pasteur or Semmelweis. The fact that Katalin Karikó and Drew Weissman were awarded the Nobel Prize in Physiology or Medicine last year for their significant contribution to this success means that we can still appreciate heroism.

Throughout history, achievements of science and technology have saved human lives and we justly consider their authors as heroes and heroines nowadays.

## The race of life: knowledge versus ignorance

But a major problem of our time is that the principles and mechanisms, by which the vaccine works, were beyond the understanding of ordinary, lay people; moreover, this gap in knowledge did not motivate a large part of the population to educate themselves. *Vice versa*. Many have quickly absorbed the lies and hoaxes spread through the internet. As a result, we were not only suffering from a biological pathogen but also from an information pathogen. Or rather, a disinformation pathogen.

… we were not only suffering from a biological pathogen but also from an information pathogen. Or rather, a disinformation pathogen.

Nevertheless, with the help of science, we have finally coped with the Covid-19 pandemic. But more pandemics are yet to come, and our reluctance to learn, combined with our willingness to be deceived will kill a large number of people again. In addition, there are threats much more difficult ahead of us, wherein we will not have so much time and chances to recover: the global climate crisis, the destruction of ecosystems, the loss of biodiversity. Happily, science offers solutions too; miserably, the scientific solutions are once again running against a wall of misunderstanding and antipathy.

But more pandemics are yet to come, and our reluctance to learn, combined with our willingness to be deceived will kill a large number of people again.

For example, to reduce the problem of greenhouse gases amplifying global warming, modern science suggests using energy from nuclear power plants and renewable sources, especially geothermal, which has a huge potential as a source of clean energy (Islam et al, 2022). But the right orientation in this issue requires mental skills higher than merely knowing that wood burns. And so, we keep pressing the pedal of fossil fuels, the concentration of greenhouse gases in the atmosphere is constantly increasing, and the Earth is set to go beyond the red line.

Another example: with its Western-style eating habits, humankind today negatively affects not only its own health, but that of our entire planet. It is called overconsumption. Since the Agricultural Revolution, the human population has grown exponentially, and it is projected to reach 10 billion by 2050. If the majority of the Earth’s population stays with the same menu, according to the findings of the EAT-Lancet commission (Willett, Rockstrom et al, 2019), the demands for our sustenance will exceed the possibilities of the Earth to feed us.

Again, science shows a way out: to consume energetically and ecologically less demanding food, for example edible insects (Stull and Weir, 2023), which provide complete nutrition and at the same time require much less natural resources compared to meat production. In parallel, the recommended dietary changes must be accompanied with the elimination of food waste at the global level. This will require cooperation, the likes of which *Homo sapiens* has not experienced since the dawn of the species. Let his selfish will to be fit and live longer be a motivation for this errand. It would be a win-win situation—for a human consumer, but also for Earth. But, seeing in whose hands the responsibility for global cooperation and the state of the planet is today gives rise to a certain scepticism since it is easier to fill our stomach with a burger and not worry too much about the global problems we cause thereby.

The most urgent threat is the loss of biodiversity (Loreau et al, 2022). During the past century, thousands of vertebrate species have died out (Ceballos et al, 2020); insect species are disappearing even faster—in a hundred years, not even a fly may fly on our planet (Rhodes, 2019). The consequences for humanity will be swift and devastating, since the truth is that humans depend on insects and not the other way around. Metaphorically: “I am creation’s crown, but frankly, I fear that creation will barely notice when I’m gone”.

The invention of the artificial intelligence (AI) offers some hope. In a recent article, Muller et al (2023) describe how they recorded the sounds of the tropical forest in Ecuador in various places—in areas untouched by logging, in logging sites and in new-grown forest—and used AI to select the voices of critically endangered tropical birds. It is a non-invasive research method as the scientists only placed microphones in the forest. The same work is done with the help of AI to identify pangolins in Uganda, gorillas in Gabon or orangutans in Malaysia (Thompson, 2023).

In addition, AI already helps doctors make more accurate diagnoses (Rodman et al, 2023), develop new antibiotics (Stokes et al, 2020) or antibodies (Bennett, 2024; Watson et al, 2023). Furthermore, AI can save not only nature, human lives, but also old forgotten songs—last year, AI extracted John Lennon’s voice from a low-quality tape recording of the song *Now and Then* recorded in the 1970s.

## A prescription for disaster

But will humans as such be able to handle AI carefully and usefully? Why not use it to falsify the voice of my political opponent or create any other kind of ‘deep fake’? It’s easier and much more rewarding than protecting bird species which most people do not care about anyway. In Slovakia, a few weeks before the release of the new Beatles song, a falsified recording of a journalist and a politician appeared on social networks. Instead of deep learning—deep fake.

“Science does not have a moral dimension. It is like a knife. If you give it to a surgeon or a murderer, each will use it differently,” said Werner von Braun (Daempfle, 2012). As long as it is a knife, only one person is in danger. But there are also tools that can take hundreds or thousands of lives or destroy the environment: nuclear fission, toxic chemicals, bioterrorism, or other nefarious applications of science and technologies. The rocket designer von Braun knew exactly what he was talking about: his invention enabled mankind to go to the Moon, but also into the grave. And the problem multiplies when tools of perfection fall in the hands of fools.

**Figure 1:**
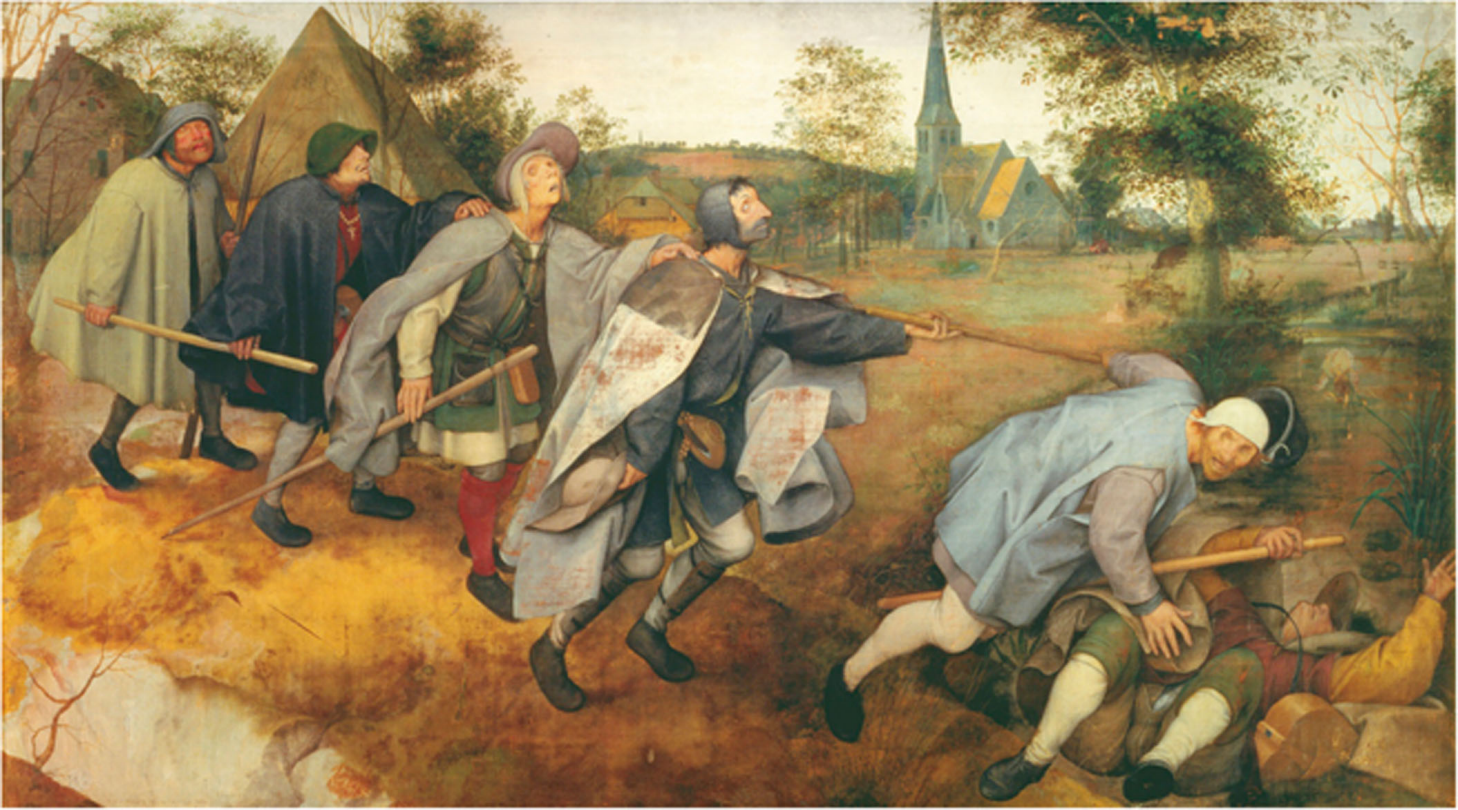
The Blind Leading the Blind by Pieter Bruegel the Elder (1568). Tempera on Canvas. Wikimedia/Public Domain.

Just as the Beatles’ first hit, *Love Me Do*, heralded a new era that culminated in humanity’s arrival on the Moon, their last single, *Now and Then*, heralds a time in which humanity—with a little help from AI—will fight for its survival on Earth. It will not be easy, because there is a strong opponent: our ignorance. The American astronomer Carl Sagan drew attention to this simple fact decades ago: “We’ve arranged a global civilization in which most crucial elements profoundly depend on science and technology. We have also arranged things so that almost no one understands science and technology. This is a prescription for disaster. We might get away with it for a while, but sooner or later this combustible mixture of ignorance and power is going to blow up in our faces” (Sagan, 1995; Fig. 1).

## The criminal life of disinformation

Is human ignorance the genius of deep crime as described by Poe in his short story? If we are fooled by a fictional story so much that we write letters to Sherlock Holmes, 221b Baker Street, London, UK, it is a bit silly and amusing. However, as soon as someone knowingly lies with the intent to steal or kill, a lie, hoax or disinformation becomes a crime.

… as soon as someone lies with the intent to steal or kill, a lie, hoax or disinformation becomes a crime.

The semiotician Umberto Eco described with a fascinating precision and clarity in one of his published lectures from Harvard University, a criminal nature of disinformation, namely the Protocols of the Elders of Zion (Eco, 1998). Eco discovered almost twenty different sources, all products of mere human imagination from various historical periods—from stories about the mysterious Templar orders, Rosicrucians and Freemasons, to romantic French novels of the 19th century. At the beginning of the 20th century, the Protocols were published in official publishing houses. A few years later, a future German chancellor referred to them in his book when he proposed the final solution to the Jewish question. The result was the Shoah, the greatest crime of human history. Such is the deadly nature of disinformation.

As noted by Poe in his clairvoyant short story *The Imp of the Perverse*: “We stand upon the brink of a precipice. We peer into the abyss—we grow sick and dizzy. Our first impulse is to shrink from the danger. Unaccountably we remain. By slow degrees, our sickness and dizziness and horror become merged in a cloud of unnamable feeling… It is merely the idea of what would be our sensations during the sweeping precipitancy of a fall from such a height… To indulge, for a moment, in any attempt at thought, is to be inevitably lost… If there be no friendly arm to check us, or if we fail in a sudden effort to prostrate ourselves backward from the abyss, we plunge, and are destroyed”.

Pushing someone into the abyss of disinformation is a crime. To succumb even for a moment to the emptiness of disinformation is a perversion. Science may be the friendly arm to save us. But to do that, science has to win the race against ignorance. And that is not an easy challenge in today’s world.

## Remember the Narts

The fate of our civilization resembles the sagas of the Narts, a folk of semigods from the Caucasian myths. They were not afraid of anything and anyone in the world and they were not afraid of their gods either. They always found a way out of every trial imposed upon them. We are also able to get out of any mess, by virtue of science, which, however, as Sagan has noted, almost no one understands. That’s why we will end up like the Narts: “…they dug their own grave and laid themselves in it.” If we don’t want to end up like them, if we want to continue our story on Earth, we need to understand modern science, just as we long ago learned how to handle fire, the greatest of human inventions. We must live up to the meaning of the name we choose for ourselves: *Homo sapiens*.

… if we want to continue our story on Earth, we need to understand modern science, just as we long ago learned how to handle fire, the greatest of human inventions.

In order to do it, we must become heroes. Today, it is easy. Each of us can save many lives, and we don’t have to jump into a troubled water to save a drowning child, rush into a burning house, or discover a magic cure despite the ridicule heaped upon us. And we don’t have to avoid “people and other foul things” like the young lord from Wodehouse’s short story.

It is enough to resist the imp of perversion, that is, to not believe disinformation, to not share it, and to expel from our community criminals who produce and spread it. It is enough if we are considerate of each other and to our environment. If we succeed, perhaps future school children will include us into their lists of heroes, who, in the post-pandemic era, looked into the abyss but did not get scared and did not jump into it.

It is enough to resist the imp of perversion, that is, to not believe disinformation, to not share it, and to expel from our community criminals who produce and spread it.

### Supplementary information

Peer Review File
