# Supplementary material for: Piccadilly full of people and other foul things: On heroism and perversion in the post-pandemic era
Source: EMBO Rep. 2024 Apr 26;25(6):2520–4. doi: 10.1038/s44319-024-00143-4 (PMC11169519; doi:10.1038/s44319-024-00143-4)
Supplement: Supplementary file 1 — Peer Review File [file 44319_2024_143_MOESM1_ESM.pdf]

EMBO reports

Piccadilly full of people and other foul things. On heroism and perversion in the post-pandemic era

Vladimir Leksa

Corresponding author(s): Vladimir Leksa (vladoleksa@gmail.com)

Review Timeline:

Submission Date:	23rd Mar 24
Accepted:	12th Apr 24

Editor: Holger Breithaupt

Transaction Report: This article was editorially reviewed.
